# Progress in the Preparation of Functional and (Bio)Degradable Polymers via Living Polymerizations

**DOI:** 10.3390/ijms21249581

**Published:** 2020-12-16

**Authors:** Si-Ting Lin, Chung-Chi Wang, Chi-Jung Chang, Yasuyuki Nakamura, Kun-Yi Andrew Lin, Chih-Feng Huang

**Affiliations:** 1 Department of Chemical Engineering, i-Center for Advanced Science and Technology (iCAST), National Chung Hsing University, Taichung 402-27, Taiwan; eclipes3625@gmail.com; 2 Division of Cardiovascular Surgery, Veterans General Hospital, Taichung 407-05, Taiwan; chungchi@vghtc.gov.tw; 3 Department of Chemical Engineering, Feng Chia University, 100 Wenhwa Road, Seatwen District, Taichung 40724, Taiwan; changcj@fcu.edu.tw; 4 Data-Driven Polymer Design Group, Research and Services Division of Materials Data and Integrated System (MaDIS), National Institute for Materials Science, Tsukuba 305-0047, Japan; 5 Department of Environmental Engineering, Innovation and Development Center of Sustainable Agriculture & Research Center of Sustainable Energy and Nanotechnology, i-Center for Advanced Science and Technology (iCAST), National Chung Hsing University, Taichung 402-27, Taiwan

**Keywords:** aliphatic polyesters, aliphatic polycarbonates, ring-opening radical polymerizations, simultaneous step- and chain-growth polymerizations, atom transfer radical polyadditions

## Abstract

This review presents the latest developments in (bio)degradable approaches and functional aliphatic polyesters and polycarbonates prepared by typical ring-opening polymerization (ROP) of lactones and trimethylene carbonates. It also considers several recent innovative synthetic methods including radical ring-opening polymerization (RROP), atom transfer radical polyaddition (ATRPA), and simultaneous chain- and step-growth radical polymerization (SCSRP) that produce aliphatic polyesters. With regard to (bio)degradable approaches, we have summarized several representative cleavable linkages that make it possible to obtain cleavable polymers. In the section on functional aliphatic polyesters, we explore the syntheses of specific functional lactones, which can be performed by ring-opening copolymerization of typical lactone/lactide monomers. Last but not the least, in the recent innovative methods section, three interesting synthetic methodologies, RROP, ATRPA, and SCSRP are discussed in detail with regard to their reaction mechanisms and polymer functionalities.

## 1. Introduction

**Scheme 1:**
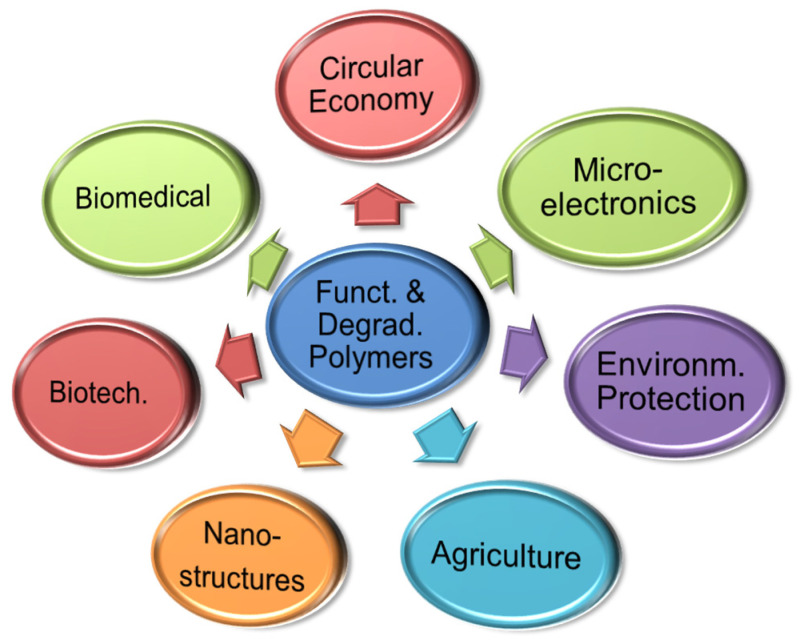
Representative applications of functional and degradable polymers.

(Bio)degradable plastics contain degradable units that include several components including at least one initiator, monomer, and cross-linker [1]. As demonstrated in Scheme 1, numerous studies and reviews have reported on degradable or cleavable structural designs and their wide range of application in biomedicine, biotechnology, agriculture, environmental protection and microelectronics. In synthetic degradable polymers, the most prevalent degradable units are ester and disulfide linkages and the other groups include hemiacetal ester, trithiocarbonate, retro-Diels–Alder, carbonate, amino-ester, thio-ester, acetal, olefin, *ortho*-nitrobenzyl ester, and so on. Table 1 presents the cleavable units and their corresponding cleavage methods/agents. Among the cleavable (bio)polymers, aliphatic polyesters, including the well-known FDA-approved poly(ε-caprolactone) (PCL) and polylactide (PLA) have attracted significant attention, not only due to their high (bio)degradability and biocompatibility but also their cost-effect features [2]. Several simple approaches to functionalized polyester-based copolymers have been demonstrated through the copolymerization of epoxides and cyclic anhydrides [3,4,5], Passerini reactions [6,7,8], or Baylis–Hillman reactions [9,10]. In this review, we have mainly focused on representative studies of functional aliphatic polyesters and several new types of degradable polyesters and polycarbonates. We start by examining innovative synthetic methods, including the design of functional lactone (f-lactone) and trimethylene carbonate monomers, radical ring-opening polymerization (RROP), atom transfer radical polyaddition (ATRPA), and simultaneous chain- and step-growth radical polymerization (SCSRP) and investigate their degradable properties and related applications.

## 2. Synthesis of Functional Aliphatic Polyesters and Polycarbonates by Ring-Opening Polymerization (ROP)

**Scheme 2:**
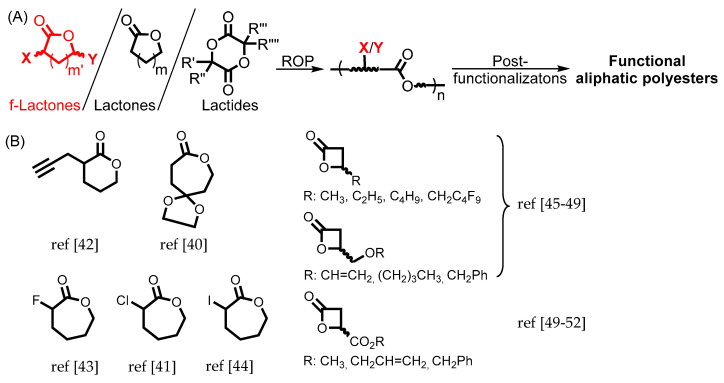
(**A**) Synthesis of functional aliphatic polyesters (f-Aps) via ring-opening polymerization (ROP). (**B**) Examples of f-lactones to attain f-APs (α-propargyl-δ-valerolactone [42]; 5-ethylene ketal ε-caprolactone (m’ = 3, X = H, Y = ketal) [40]; α-fluoro-ε-caprolactone (m’ = 3, X = F, Y = H) [43]; α-chloro-ε-caprolactone (m’ = 3, X = Cl, Y = H) [41]; α-iodo-ε-caprolactone (m’ = 3, X = I, Y = H) [44]; *racemic* 4-alkyl methylene-β-propiolactones (m’ = 0, X = H, Y = -CH_3_, -C_2_H_5_, -C_4_H_9_, -CH_2_C_4_F_9_) and *racemic* 4-alkoxymethylene-β-propiolactones (m’ = 0, X = H, Y = -OCH = CH_2_, -O(CH_2_)_3_CH_3_, -OCH_2_Ph) [45,46,47,48,49]; alkyl β-malolactonates (m’ = 0, X = H, Y = -COOCH_3_, -COOCH_2_CH = CH_2_, -COOCH_2_Ph) [49,50,51,52]).

Ring-opening polymerization (ROP) is one of the most widely used methods for the syntheses of aliphatic polyesters [31]. In the presence of the initiator or the catalyst, lactones and lactides can be polymerized efficiently through the fragmentation of the ring, typically in an anionic pathway under mild reaction conditions, which produces aliphatic polyesters with highly (bio)degradable and biocompatible features. This analog of aliphatic polyesters can be reacted with the chain-end hydroxyl group(s) to perform further functionalization, modifications, or chain extensions. Thus, the limited numbers of functionalizable end(s) thus can be anticipated [32,33,34]. However, rendering a variety of functional groups on aliphatic polyester backbones remains challenging since ROP cannot usually tolerate high-polar groups [35,36,37,38,39]. As shown in Scheme 2A, (co)polymerization of functional lactones (f-lactones) and lactones/lactides provides a simple synthetic approach to render groups that are less polar but compatible with typical ROP catalysts. Numerous studies on the synthesis of functional aliphatic polyesters (f-APs) are listed in Scheme 2B. Most notably, Jérôme et al. [40] reported the first case of controlled/living ROP of 5-ethylene ketal ε-caprolactone to produce well-defined PCL with cleavable pendant groups (*M*_n_ = ca. 7000 and *M*_w_/*M*_n_ = 1.15). PCL backbones with hydroxyl pendant groups can be quantitatively obtained through an efficient deacetalization reaction. The novel amphiphilic PCL can also form stable and homogeneous colloidal solutions in water. The study presented the pioneering idea of functional cyclic monomer designs and explored the synthesis of f-APs and their application in aqueous solutions. In another example of the synthesis of f-AP and their related applications, Chang et al. [41] reported the synthesis of f-APs possessing pendant (*pen*) chlorides via ROP of α-chloro-ε-caprolactone and ε-caprolactone (i.e., PCL–*pen*–(n Cl), n = 10, *M*_n_ = ca. 17,800 and *M*_w_/*M*_n_ = 1.5). The pendent chlorides can subsequently be converted to azides and used in Cu(I)-catalyzed alkyne-azide cycloaddition reactions to graft nucleobase hydrogen bonding units, i.e., uracil (U)/adenine (A) along the PCL backbones. Mediation by multiple hydrogen bonding units results in two types of complementary macromolecules that can form stable and reversible physical crosslinking networks. Further evaluation by L929 cell cytotoxicity tests revealed that the PCL-based supramolecular networks possess excellent biocompatible properties. This innovative study demonstrates the preparation of physically crosslinked and mechanically stable PCL materials with excellent biocompatible properties that have potential for biomedical engineering applications.

On the other hand, β-propiolactone derivatives can be regarded as a renewable type monomer obtained by effective and eco-friendly carbonylation of *racemic* epoxides [53]. Interestingly, Thomas et al. [54] addressed the polymerization mechanism of “syndio-control” from stereo-monomers. For example, ROP of *racemic* 4-alkyl-β-propiolactones (*rac*BPL-Rs) with various alkyl groups (e.g., -R: -CH_3_, -C_2_H_5_, -C_4_H_9_, -CH_2_C_4_F_9_) produces functional polyhydroxyalkanoates (PHAs) with well controlled tacticity. As revealed from the in situ ^1^H NMR measurements, the formation of a highly alternating PHA was achieved. The elegant syndio-controlled ROP provides a new type of promising (bio)degradable aliphatic polyesters. With the design of effective and specific catalysts, Carpentier et al. [45,46,47,49] recently reported an elegant strategy that uses rare-earth complexes that incorporate dianionic diamino-oramino-alkoxy-bis(phenolate) ligands to obtain novel PHA copolymers. Basically, a simple and effective chain-end syndio-control mechanism is used, which results in the stereo-selective ROP of chiral β-lactones. For example, the ring-opening copolymerization of *racemic* 4-alkoxymethylene-β-propiolactones (*rac*BPL-ORs) was studied (-OR: -OCH_2_CH = CH_2_, -OCH_3_, -OCH_2_Ph, -OSi(CH_3_)_2_C(CH_3_)_3_). The results revealed stereo-control via the catalysts and specific alternative poly(3-hydroxybutyrate) (PHB) copolymers of P[(HB-OR^1^)-*alt*-(HB-OR^2^)] with various alkoxy groups (e.g., -OR^1^: -OCH_2_CH = CH_2_; -OR^2^: -OCH_3_) were obtained. The main factors for achieving a high degree of alternation include the use of: (i) a highly syndio-selective catalyst; and (ii) a proper ratio of (*R*)-BPL-OR^1^/(*S*)-BPL-OR^2^ monomers. Accordingly, we can expect the formation of novel stereo-complexes through the blending of the resulting enantiomorphic polyesters.

**Scheme 3:**
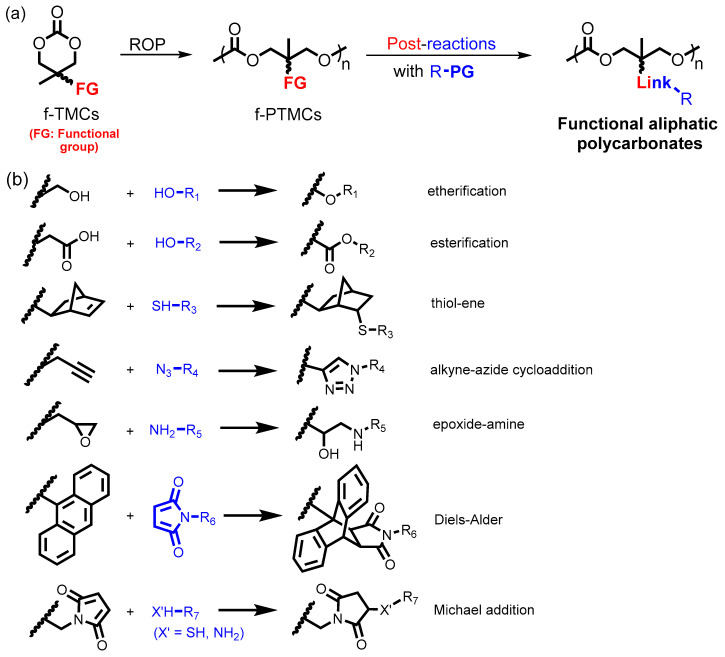
(**a**) General structures of f-TMCs with functional groups (FGs) on trimethylene carbonates (TMCs) and post-reactions with R-PG (macro)molecules. (**b**) Examples of post-reactions between f-PTMCs and R-PG (macro)molecules through the formation of new linkages (i.e., forming “Link”).

Besides the well-known aliphatic polyesters, another family of emerging, highly (bio)degradable and biocompatible aliphatic polycarbonates, poly(trimethylene carbonate) (PTMC), have also been extensively investigated [55]. Being the starting material for trimethylene carbonate (TMC), 1,3-propanediol can be acquired from the degradation of natural carbohydrates or ring-closure carbonylation of carbon dioxide [56]. The analog of TMC monomers is thus referred to as a renewable resource. As shown in Scheme 3a, f-PTMCs can be synthesized by ROP of f-TMCs through either typical organometallic catalysts or organo-catalysts [57]. As illustrated in Scheme 3b, several representative functional groups (FG) on the TMC ring are addressed, including (i) OH, (ii) COOH, (iii) allyl/SH, (iv) propargyl, (v) epoxide, (vi) norbornene, (vii) maleimide, and so on. In order to further graft specific (macro)molecules, post-reactions between FG and PG can be performed via (i) etherification, (ii) esterification, (iii) thiol-ene, (iv) alkyne–azide cycloaddition, (v) epoxide–amine, (vi) Diels–Alder, (vii) Michael addition, and so forth. For example, Harth et al. [58] reported the synthesis of PTMC-based hydrogels with various crosslinking reagents. They first synthesized PTMCs with pendant functional groups of both ethyl ester (i.e., PTMC-*pen*-(O)COEt) and allylic ester (i.e., PTMC-*pen*-(O)COCH_2_=). Subsequently, thio-ene click reactions of the (PTMC-=) and (HS-(EG)_n_-SH) (EG: ethylene glycol; n = 1 or 35) were conducted to obtain PTMC-crosslinked hydrogels. A polyol of branched polyglycidol (PGY) and a transesterification catalyst of zinc acetate (Zn(OAc)_2_) were further introduced into the hydrogels. Interestingly, the composite underwent a chemical self-modification from the PTMC/PGY/(Zn(OAc)_2_) hydrogels at high temperature (ca. 120 °C) on the basis of dynamic covalent bonds. Accordingly, the attractive renewable feature of f-TMC monomers and their ability to render diverse pendant functional (macro)molecules on f-PTMCs has led to very high expectations for their application to various practical uses.

## 3. Synthesis of Aliphatic Polyesters by Radical Ring-Opening Polymerization (RROP)

Polyesters are typically synthesized by either ROP of lactones/lactides [31] or polycondensation of hydroxyl and acid monomers [59]. In the 1980s, Bailey et al. [60,61,62,63,64] reported the pioneering cases of free radical ring-opening polymerizations (RROPs) of specific cyclic ketene acetal (CKA) and cyclic acrylate (CA) monomers initiated by conventional thermal initiators (i.e., peroxide and azo compounds). These studies established an innovative approach for producing a variety of functional polyesters on the basis of radical chemistry. Thereafter in the 1990s, Endo et al. [65,66,67] and Rizzardo et al. [68] designed a series of specific vinylcyclopropane (VCP) and cyclic allylic sulfide (CAS) monomers, respectively, to produce f-APs as well. RROP of VCPs rendered ester linkages in the backbone and also improved the introduction of other functionalities into the backbone (e.g., olefin and phenyl) and at the pendent sides (e.g., benzyl and ethylene ketal) (*M*_n_ = 22,000 and *M*_w_/*M*_n_ = 2.05). In the case of the RROP of CASs, polyester backbones with sulfide linkages and pendant double bonds were obtained (*M*_n_ = 46,200 and *M*_w_/*M*_n_ = 2.3).

**Scheme 4:**
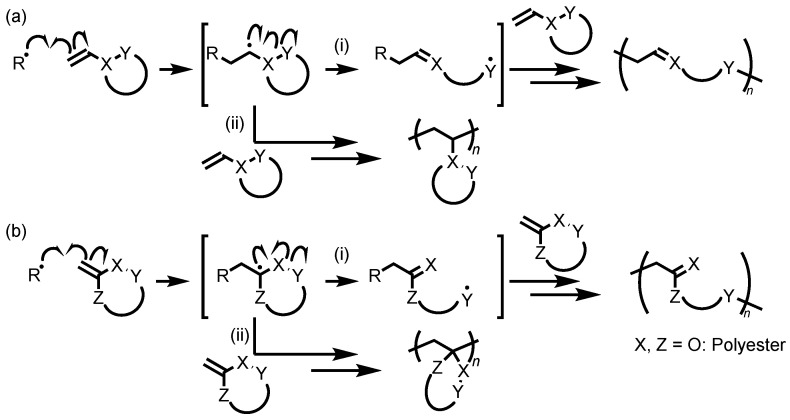
Mechanism of radical ROP (RROP) for vinyl type (**a**) and *exo*-methylene type (**b**) monomers. For both monomer types, two possible polymerization mechanisms exist: (i) RROP and (ii) vinyl polymerization.

The monomers for RROP are classified into two types: the vinyl type such as VCP and the *exo*-methylene type such as CKA. In the RROP mechanism of both monomers, a radical species is added to the carbon–carbon double bond and thus generates a carbon-centered radical, then the radical ring-opening reaction generates a new carbon-centered radical via the cleavage of a carbon–carbon bond or a carbon-heteroatom bond (Scheme 4). The polymerization of these monomers proceeds inherently via the RROP mechanism or a conventional vinyl polymerization mechanism. Only RROP provides an ester structure in the polymer main chain, thus it is essential to use monomers that have high RROP selectivity. Kinetic and thermodynamic factors are required for the selectivity: (i) the ring-opening reaction is accelerated due to the strain of the ring; and (ii) the resulting new carbon-centered radical is stabilized and/or the ring-opening reaction involves thermodynamically favored isomerization of the functional group.

**Scheme 5:**
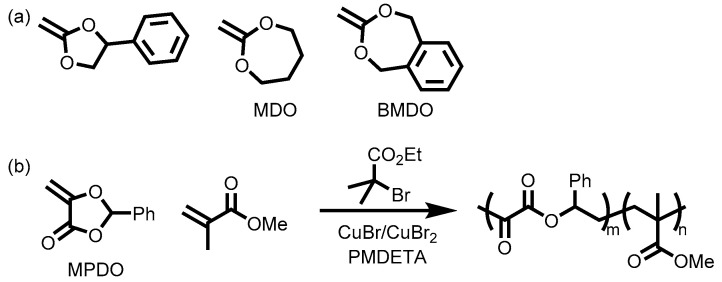
(**a**) Representative cyclic ketene acetal (CKAs) with high or exclusive RROP selectivity. (**b**) An example of atom transfer-mediated RROP (AT-RROP) for the synthesis of P(MMA-*co*-MPDO) copolymers [73,74].

Scheme 5a displays representative CKAs with high or exclusive RROP selectivity. One of the most attractive features of RROP of CKA is the copolymerization with conventional vinyl monomers including (meth)acrylates, styrenes, vinyl pyridine, vinyl acetate, and *N*-vinylpyrrolidone, which can readily provide various (bio)degradable f-PE. The size of the ring of CKA is one of the major factors controlling the selectivity in the mechanism. CKA with strained rings, for example, the 7 and 8-membered ones are more favorable in the RROP compared to the stable 5 and 6-membered ones. Namely, the polymerization of the 7-membered ring monomer MDO exclusively provides polyester via the selective RROP; however, the selectivity of the RROP mechanism of the corresponding 6-membered ring monomer 2-methylene-1,3-dioxane decreases to about 50%. Another important structural factor is the introduction of a stabilizing group for the carbon-centered radical generated by the ring-opening, which are typically phenyl or alkyl groups. While the ring-opening occurs from both sides of an acetal ring, the introduction of a radical stabilizing group regulates the side of the ring-opening that has the group. In addition, the relative reactivity between CKA and the vinyl monomer is critical in the copolymerization to prepare f-PE. Because the olefin of CKA is very electron-rich, the reaction with propagating radicals formed from common vinyl monomers, which are nucleophilic or amphiphilic, is relatively unfavored [69]. Therefore, the reactivity ratio in the copolymerization of CKA and vinyl monomers is often r_CKA_ << 1 < r_vinyl_, which impedes the incorporation of the CKA monomer and causes a deviation between the composition of polymer from the monomer feeding ratio. For example, in the copolymerization of MDO and MMA, the reactivity ratios are r_MDO_ = 0.057 and r_MMA_ = 34.12 [70], and the composition of MDO in the resulting polymer is 4% (polymerization at 40 °C) or 30% (at 120 °C) from copolymerization with a monomer feeding ratio of MDO/MMA = 54/46 or 50/50, respectively. On the other hand, the copolymerization of CKA with vinyl acetate (VAc) undergoes in an almost random manner (i.e., more statistical distribution of monomers in the chain), which is confirmed by r_CKA_ = 0.93 and r_VAc_ = 1.71 in the copolymerization with MDO [71], and this provides a copolymer with a homogeneous composition of CKA and VAc that is similar to the monomer feeding ratio. This feature is not limited to VAc, and other vinyl carboxylate monomers also give a copolymer with CKA in an almost random manner. Additionally, the copolymerization of vinyl bromobutanoate and MDO [72], with post-modification through alkyne-azide cycloaddition results in PEG-grafted degradable polyester.

Recently, interesting progress in the scope of copolymerization has been reported. Guillaneuf et al. [75] reported the copolymerization of vinyl ether and MDO in a highly random manner. The composition of monomers during the polymerization reaction was found to follow the initial feeding ratio of the monomers. The copolymerization reactivity ratio was r_MDO_ = 0.73 and r_vinyl ether_ = 1.61, which is consistent with of the theoretical calculation of the reaction rate for the α-oxyethyl radical and MDO. The highly random manner could also be related to the fact that vinyl ethers are not homopolymerized by the radical polymerization. The synthetic benefit of vinyl ether for f-PE is the ready availability of various functional monomers, and indeed, Cl, oligo(ethylene glycol) and terminal alkene functionalized vinyl ethers have been shown to give copolymers with MDO. These copolymers were further modified to obtain a fluorescent probe functionalized polymer and a degradable elastomer. Another recent example is the copolymerization of BMDO and maleimide reported by Sumerlin [76]. The copolymerization proceeded with the quantitative ring-opening of BMDO to the ester and in a highly alternating manner. The copolymer was readily functionalized by utilizing *N*-substituent of maleimide, and the alternating structure might be suitable for fast degradation to low molecular weight fragments. Interestingly, although the alternative copolymerization of other CKAs such as MDO and maleimide has been reported, the selectivity of the ring-opening of CKA was not enough, which indicates that the appropriate combination of monomer and the reactivity is quite important in designing the CKA copolymer as a f-PE.

In conventional free radical polymerization, radicals are produced in the initiation step (*k*_d_ = ca. 10^−4^–10^−6^ s^−1^) through the decomposition of thermal or photo-initiators. Subsequently, a moderate-to-fast chain propagation (depending on the monomers: *k*_p_ = ca. 10^2^–10^4^ M^−1^s^−1^) and very fast radical terminations (*k*_t_ = ca. 10^6^–10^8^ M^−1^s^−1^) occur [77]. Due to the irreversible radical generating step, rather high concentrations of the unstable species cause a significant number of terminations, which leads to broad molecular weight distributions and uncontrollable kinetics. Although RROP provides an alternative approach to the synthesis of f-APs, the RROP method has to meet some application demands that require a narrow range of molecular weight distributions. In the mid-1990s, reversible-deactivation radical polymerization (RDRP) was discovered and it is still being developed in academia and industry [78]. In RDRP, reagents of dormant molecules and regulators (which act as activators and deactivators) are necessarily present. The key to achieving controlled/living polymerization is to follow a number of general steps [78]: (i) the initiating rate of the reaction between the activators and dormant molecules should be faster than that of the chain propagating rate; (ii) meanwhile, deactivators and active radicals are produced in order to proceed with certain monomer additions; (iii) the concentration of the (macro)radicals is quickly deactivated by deactivators; and (iv) meanwhile, activators and dormant (macro)molecules are reversibly generated so that another cycle can be conducted starting from step (i). In a controlled/living polymerization, the concentrations of active radicals should remain low in order to achieve the suppression of termination reactions and so that all polymer chains can grow evenly and consecutively. A homogeneous dispersion of a regulator in the polymerization mixture provides an effectively controlled/living process. However, the initiating sites (i.e., active centers) can be either homogeneously dispersed in the polymerization mixture or attached to various heterogeneous surfaces of silicon, metals, and plastics (e.g., wafers, plastic tubes, porous materials, (nano)fibers, (nano)particles, etc.).

Among RDRPs, the most widely used techniques include atom transfer radical polymerization (ATRP) [79,80,81,82], nitroxide-mediated radical polymerization (NMRP) [83], and reversible addition-fragmentation chain transfer (RAFT) polymerization [84,85,86,87]. Accordingly, the diverse techniques of RDRPs have been introduced to RROP systems to obtain well-defined (co)polymers with ester linkages. For instance, Matyjaszewski et al. [73] carried out the atom transfer-mediated RROP (AT-RROP) of CA or CKA monomers with typical MMA or St monomers. Scheme 5b shows an example of the AT-RROP of MMA and MPDO to provide hydrolysable P(MMA-*co*-MPDO) random copolymers (*M*_n_ = ca. 16,300 and *M*_w_/*M*_n_ = 1.31). Interestingly, repeating units of ring-opened (i.e., forming α-ketoester linkages) and non-ring-opened (i.e., proceeding 1,2-vinyl additions) from the MPDO monomer were attained. Similar results were found in comparisons of AT-RROP and conventional RROP methods. Some other AT-RROPs of CA or CKA type monomers with typical vinyl monomers have also been demonstrated [74,88,89,90,91,92,93,94,95,96]. Accordingly, nitroxide-mediated RROP (NM-RROP) [97,98] and reversible addition-fragmentation chain transfer-mediated RROP (RAFT-RROP) [99,100] have also been effectively applied to obtain well-defined polymers with ester linkages in the backbone. Table 2 summarizes the monomers, polymer structures, their related synthetic methods, and applications on the basis of a RROP approach.

## 4. Synthesis of Degradable Polyesters by Atom Transfer Radical Polyaddition (ATRPA)

The normal ATRP and its derivative techniques [80] are based on atom transfer radical addition (ATRA) [101,102]. Recently, extensions of multi-step ATRA created a novel analog of aliphatic polyesters with functional groups on their polymer backbones that can be obtained through manipulation of the different activation/deactivation rate constants of the inimers (i.e., initiator and monomer). In 1997, preliminary research on the preparation of aliphatic polyesters through ATRP of AB-type inimers was reported by Matyjaszewski et al. [103,104]. The studies addressed the possibility of obtaining aliphatic polyesters during atom transfer-induced radical self-condensing vinyl polymerization (ATR-SCVP) of aliphatic ester type inimers. Through the ATR-SCVP of 2-((2-bromopropionyl)oxy)ethyl acrylate (BPEA), topological (hyper)branched polymers comprising both aliphatic polyester and polyacrylates structures were attained. Later, Li et al. coined the term, “atom transfer radical polyaddition” (ATRPA), for the synthesis of perfect linear aliphatic polyesters.

**Scheme 6:**
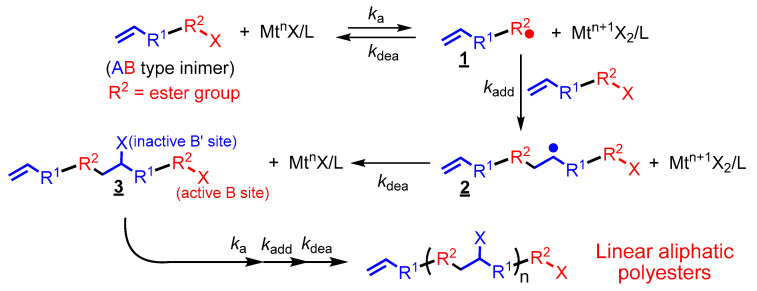
General mechanism of transition metal catalyzed atom transfer radical polyaddition (ATRPA).

Scheme 6 demonstrates the general mechanism of ATRPA, which provides perfect linear aliphatic polyesters (i.e., the R^2^ linkage comprising the ester group). The first example of perfect control ATRPA was demonstrated by Kamigaito et al. in 2007 [105]. Through the design of a specific AB type inimer (e.g., allyl 2-chloropropanoate) with a reactive C–Cl bond (i.e., site B), they developed a novel and radical polyaddition method perfectly controlled by transition metals. The specific inimer can be activated by a lower-oxidation state transition metal (e.g., Cu(I), Ru(II), Fe(II), etc.) to form a radical species (i.e., species **1**) and react with a double bond (i.e., site A) of another inimer. More importantly, the newly formed single addition radical species (i.e., species **2**) can be deactivated by a higher-oxidation state transition metal (e.g., Cu(II), Ru(III), Fe(III), etc.) to obtain a dimer that possesses extremely inactive pendant C–Cl bonds (i.e., site B’ on species **3**). By perfectly and slowly repeating the single addition of inimers, linear aliphatic polyesters can be obtained [106,107]. Li et al. also designed an AB type inimer (i.e., (4-vinylbenzyl 2-bromo-2-isobutyrate (VBBiB)) [108] or AA/BB paired monomers, i.e., bis(styrenics)/bis(bromoisobutyrates) [109,110]. They made four critical breakthroughs on the basis of the ATRPA technique including: (i) controlling the topology from hyperbranched to linear polymers; (ii) improving the effectiveness of perfect-control ATRPA, i.e., polymerizations were reduced to a few days; (iii) rendering a variety of functional groups into the linear polymer backbone, i.e., diverse functional linkages between R^1^ and R^2^; and (iv) grafting different functional polymers onto the linear polymer backbone, i.e., through post-reactions of C–X bond. In polymerizations of VBBiB in anisole at 0 °C with a homogeneous catalyst system, for example, a step-growth trend was detected. At such a low temperature, high selectivity between the inactive B’ and active B sites can be retained, leading to the formation of linear aliphatic polyesters. In polymerizations of VBBiB in toluene at 0 °C with a heterogeneous catalyst system, the deactivation efficiency of the active B• radical (chain-)ends was insufficient, which led to fast conventional free radical polymerizations to produce linear polymers with C–C as the backbones and bromoisobutyryl as the pendant groups. Polymerizations of VBBiB in anisole at high temperatures (i.e., 20–60 °C) with a homogeneous catalyst system resulted in low selectivity between the inactive B’ and active B sites, leading to the formation of branched polymers through the mechanism of atom transfer-induced radical self-condensing vinyl polymerization (ATR-SCVP). Further, Kamigaito et al. and Li et al. utilized metal-catalyzed intermolecular radical polyadditions to design sequence-regulated vinyl polymers by exact manipulation of functionality equivalents [102,107].

Recently, Huang et al. [87,111,112] designed a highly reactive AB type inimer (i.e., 4-vinylbenzyl 2-bromo-2-phenylacetate (VBBPA)), which significantly improved the reactivity for ATRPA (i.e., polymerizations were reduced to a few hours) but kept high selectivity, to obtain linear aliphatic polyesters. Therefore, high molecular weight aliphatic polyesters (*M*_w_ = ca. 26,000 and *M*_w_/*M*_n_ = 2.09) can be effectively obtained in three hours. This significant improvement was due to two factors: (i) the activation rate of the VBBPA initiating site is much faster than that of VBBiB (i.e., *k*_a,VBBPA_/*k*_a,VBBiB_ = ca. 2 × 10^3^ at 35 °C), which results in highly reactive ATRPA; and (ii) the difference in the activation rate in the C–X groups at the chain ends and the inactive C–X groups on the backbones is extremely large (i.e., *k*_a,C–X(PVBBPA end)_/*k*_a,C–X(PVBBPA backbone)_ = ca. 3 × 10^4^ at 35 °C), which results in a highly selective ATRPA. By tracing the ATRPAs of VBBiB and VBBPA, an interesting self-degrading behavior was observed in the PVBBiB, which resulted in the formation of a five-membered-ring lactone structure (i.e., (5-(4-(bromomethyl)phenyl)dihydro-3,3-dimethylfuran-2(3*H*)-one)). In the same circumstances, the PVBBPA performed as a stable type polyester. Eventually, post-click reactions were applied to obtain amphiphilic polymer brushes (i.e., aliphatic polyesters as the backbone; hydrophilic poly(ethylene glycol) as the grafting chains). The novel polyesters possess pH-sensitive and reversible thermoresponsive behaviors [110,113]. Therefore, the development of innovative ATRPAs provides an alternative strategy for obtaining f-APs.

## 5. Synthesis of Degradable Polyesters by Simultaneous Chain- and Step-Growth Radical Polymerization (SCSRP)

**Scheme 7:**
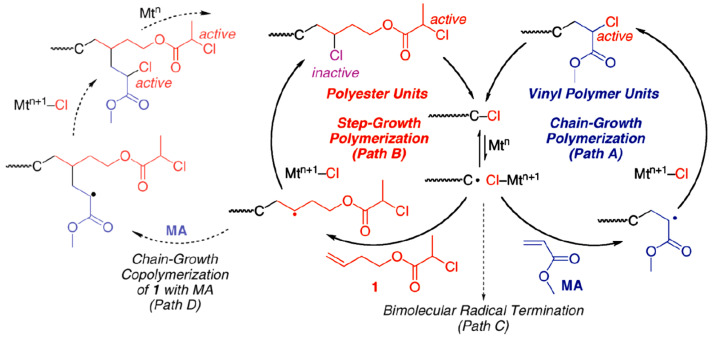
An exemplified mechanism of simultaneous chain- and step-growth radical polymerization (SCSRP) of MA and inimer **1** [96].

Kamigaito et al. developed another novel technique, which was similar but different to ATRPA, for simultaneous chain- and step-growth radical polymerization (SCSRP) [114]. As shown in Scheme 7, a common vinyl (i.e., methyl acrylate (MA)) and an inimer (i.e., compound **1**) are both present in the reaction mixture. Mediated by transition metal catalysts (e.g., Cu(I), Ru(II), Fe(II), etc.), the resulting atom transfer reactions can effectively perform simultaneous ATRP (i.e., Path A) and ATRPA (i.e., Path B) mechanisms. The proper design of the inimer (i.e., compound **1**) means that the branching reactions via the ATR-SCVP mechanism (i.e., Path D) can almost be suppressed. Eventually, novel polymer structures are obtained that are comprised of both polyvinyl and polyesters as the backbone (*M*_w_ = ca. 36,000 and *M*_w_/*M*_n_ = 2.01). That is, the chemical structures obtained via the SCSRP of vinyls and effective inimers are similar to the copolymers obtained via the RROP of vinyls and CKAs. However, the CKA monomers have very poor reactivity toward copolymerization with vinyls, which limits the introduction of ester linkage into the polyvinyl backbones. Thus, copolymers with varying compositions of polyvinyl and polyester can be effectively attained via the SCSRP technique [115,116,117]. Zhu et al. [118] performed fast and effective SCSRP of MA and ABP (i.e., allyl 2-bromopropanoate) to obtain P(MA-*co*-ABP) copolymer (*M*_w_ = ca. 5100 and *M*_w_/*M*_n_ = 1.78) with both a degradable ester group and an undegradable poly(acrylate) segment. They also identified the α-double bond at the copolymer chain end. Then, efficient thiol-ene click reactions of thiol-terminated PNIPAM (poly(*N*-isopropyl amide)) and double bond-terminated P(MA-*co*-ABP)s were performed. Serial novel block copolymers of PNIPAM-*b*-P(MA-*co*-ABP) were synthesized and these displayed thermoresponsive properties with lower critical solution temperatures (LCSTs: 34–37 °C). SCSRP successfully linked the undegradable polyvinyl (i.e., C–C linkages in the backbones) and the degradable polyester (i.e., ester groups in the backbones) to prepare functional and eco-friendly commodity plastics.

## 6. Conclusions and Outlook

In this report, we first discussed the most recent novel synthetic methods for preparing functional polyesters. Then, recent topics of interest in regard to the synthesis of polyesters, including the use of bio-originated or “sustainable” monomers, organocatalyzed ROP for polyesters, and the efficient functionalization of polyesters are summarized. Finally, recent innovations in the polymer chemistry of RROP, ATRPA, and SCSRP methodologies have created a novel series of (bio)degradable and functional polyester-containing polymers. These novel functional polyesters have great potential for application in biomedical, biotechnology, nanomaterials, microelectronics as well as contributing to a circular economy, environmental protection, and agriculture.

The development of degradable, especially biodegradable polymers with functional properties is becoming increasingly important. For example, the European Chemicals Agency recently announced a recommendation to restrict the amount of micro-plastic additives in products and there are other demands for environmental protection. Biodegradable polymers are excluded from these regulations; however, the polymers are required to reach degradability standards that are much stricter than in the past. The regulations regarding use of micro-plastics are also expected to be imposed on various (synthetic) polymer products. Therefore, polymers (as products and additives) with a wide range of functionality and sufficient value, which have low environmental impact and high (bio)degradability are highly desirable in the long run.

## Figures and Tables

**Table 1:** Cleavable units, cleaving methods/agents, and main generated group(s) after cleavage.

Name of Cleavable Unit	Cleaving Method/Agent	Generated End-Group(s) after Cleavage	Ref
Ester	acids/bases/enzymes	hydroxyl & carboxylic acid	[11]
Disulfide	DTT/GSH/Bu_3_P	thiol	[12,13,14]
Hemiacetal ester	acids/alcohols	carboxylic acid	[15,16]
Trithiocarbonate	amines	thiol	[17,18]
Carbonate	acids/bases/enzymes	hydroxyl	[19,20]
Amino-ester	acids/bases/enzymes	amine & carboxylic acid	[21,22]
Thio-ester	acids/bases	thiol & carboxylic acid	[23,24]
retro-Diels–Alder ^1^	heat	furan & maleimide	[25,26]
Acetal	acids/TFA_(g)_^2^	hydroxyl	[27]
*ortho*-Nitrobenzyl ester	UV (350 nm)	*ortho*-nitrobenzaldehyde & carboxylic acid	[28,29]
Olefin	ozone	aldehyde	[30]

^1^ An example of rDA reactions based on a pair of furan and maleimide moieties. ^2^ TFA_(g)_: vapor of trifluoroacetic acid (DTT: dithiothreitol; GSH: glutathione; Bu_3_P: tributylphosphine; TFA: trifluoroacetic acid).

**Table 2:** Summary of monomers, polymer structures, their related synthetic methods, and applications.

Monomer(s)	Polymer Structure	Synthetic Method	Application ^a^	Ref
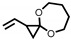	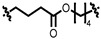	RROP	Degradable	[65]
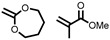	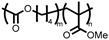	RROP	Degradable	[70]
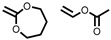	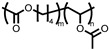	RROP	Degradable	[71]
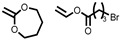	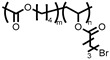	RROP	Degradable	[72]
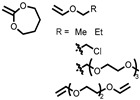	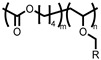	RROP	Degradable & FL ^b^ Antibacterial Degradable elastomer (via post-modifications)	[75]
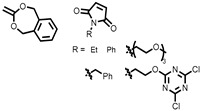	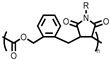	RROP	Degradable	[76]
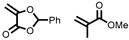	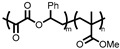	AT-RROP	Degradable	[73]

^a^ Specific applications reported in the study [75], or the degradability as the general interest of the application. ^b^ FL: fluorescent.
